# Effects of lenvatinib on glucose, cholesterol, triglycerides and estimated cardiovascular risk in patients with advanced thyroid cancer

**DOI:** 10.1007/s12020-024-04003-y

**Published:** 2024-09-17

**Authors:** E. Acitelli, A. Verrienti, M. Sponziello, V. Pecce, I. Minicocci, M. Macera, S. Barp, P. Lucia, G. Grani, C. Durante, M. Maranghi

**Affiliations:** Department of Translational and Precision Medicine, Sapienza University, Rome, Italy

**Keywords:** MKIs, Glycemia, Hyperglycemia, Dyslipidemia, Hypercholesterolemia, Hypertriglyceridemia

## Abstract

**Purpose:**

Multitarget kinase inhibitors (MKIs) are effective options in the treatment of cancer, significantly increasing the progression-free survival (PFS) of many tumors. Data about severity and prevalence of metabolic adverse events is scarce and may be significant in patients with a better survival. The aim of this study was to investigate glucose and lipids values of patients treated with lenvatinib. Secondary aims included evaluating changes in the estimated risk of cardiovascular disease and the relationship between metabolic alterations and tumor response to therapy.

**Methods:**

A retrospective pilot study on 29 patients with advanced differentiated thyroid cancer was conducted. Clinical and biochemical characteristics were collected at the day of therapy initiation and follow up. The 10-year risk of cardiovascular disease was estimated with the SCORE2 and SCORE2-OP algorithms. Tumor burden change was assessed according to the Response Evaluation Criteria in Solid Tumors (RECIST).

**Results:**

No differences in glucose values were observed. A significant increase in total cholesterol (208 ± 41 versus 245 ± 67 mg/dl), triglycerides (112 [interquartile range, 58–326] versus 157 [78–296] mg/dl), calculated LDL cholesterol (128 [66–204] versus 140 [81–308] mg/dl) and cardiovascular risk was observed from baseline to follow up. Furthermore, these parameters increase progressively with increasing tumor response to therapy.

**Conclusions:**

Despite limitations, this study shows an association between the use of lenvatinib and the development of lipid alterations in patients with advanced thyroid cancer. However, further investigation is necessary for a more comprehensive understanding of the adverse metabolic profile of MKIs.

## Introduction

Tyrosine kinase inhibitors (TKIs) have emerged as effective options in the treatment of cancer due to the demonstrated altered activation of these receptors during the development of diverse tumors [1].

Lenvatinib is a multi-target kinase inhibitor (MKI) targeting vascular endothelial growth factor receptors 1–3 (VEGFR1–3), fibroblast growth factor receptors 1–4 (FGFR-1–4), RET, c-kit, and platelet-derived growth factor receptor α (PDGFRα), approved for treatment of radioactive iodine (RAI)-refractory differentiated thyroid cancer (DTC) [2]. A pivotal randomized trial (SELECT trial), leading to the drug approval [3], compared lenvatinib with placebo: lenvatinib demonstrated a significantly improved progression-free survival (PFS) of 18.3 months. A starting dose of 24 mg/day was confirmed to be superior to 18 mg in a subsequent randomized study [4].

In real-life studies, lenvatinib achieved an overall response rate of 36–38% and a lower median PFS of 9.7–12 months [5–8]. Notably, real-life data from France involving 75 patients (in some cases pre-treated with multiple other kinase inhibitors or cytotoxic chemotherapy) showed slightly worse results [8], with a median PFS of 10 months (median overall survival [OS] was not reached). A large Japanese study (COLLECT study) focusing on OS, demonstrated a 1-year OS rate in the total population of 85.6% [9, 10].

Advanced thyroid cancer is a rare disease, but the introduction of MKIs as part of the therapy led to a significant increase in the 5-year relative survival rate [11], with some patients experiencing up to 10 years of overall survival [12]. In particular, while non-progressive radioiodine refractory DTC patients have a median OS of more than 9 years [12], multiple studies still failed in demonstrating a significant increase in OS in progressive patients treated with MKIs.

On the other hand, several adverse events (AEs) have been reported in patients treated with MKIs, including mainly hypertension, diarrhea, anorexia, weight loss, fatigue, and proteinuria [13]. Endocrine-related effects have been described as well. Beside hypothyroidism, which is a frequent complication in patients with hepatocellular carcinoma treated with lenvatinib [14], subjects treated with MKIs can also develop metabolic disorders, including diabetes (~15–40%) and dyslipidemia (~50%) [1]. Glucose and lipids alterations are not always investigated in clinical trials of MKI: a recent systematic review reported rates of hypercholesterolemia ranging from 4 to 40%, hypertriglyceridemia ranging from 2 to 86%, and hyperglycemia from 1 to 19%, with several sources of heterogeneity [15].

As stated in the 2022 ESC Guidelines on cardio-oncology, inhibition of the VEGF signaling pathway can lead to an increase in the cardiovascular risk of patients. This increase is thought to be mediated by the development of hypertension, QTc prolongation, acute vascular event, and heart failure [16].

For anti-VEGF, differently from other categories of anti-cancer drugs (such as immune checkpoint inhibitors, endocrine therapies for breast cancer, androgen deprivation therapy for prostate cancer, cyclin-dependent kinase inhibitors), possible effects on metabolic profile are not mentioned in the guidelines, due to insufficient amount of evidence [16]. Nevertheless, an increased risk of myocardial infarction (MI) is reported with these drugs [16].

In the light of these considerations, understanding the entity of possible metabolic alterations in patients treated with anti-VEGF MKIs is gaining more importance.

The aim of the study was to assess the prevalence and severity of metabolic alterations in patients with advanced differentiated thyroid cancer before and after starting lenvatinib.

Secondary aims included evaluating changes in the estimated risk of cardiovascular events after therapy initiation and studying the association between metabolic profile and tumor response to therapy assessed according to the RECIST (Response Evaluation Criteria in Solid Tumors) classification system.

## Materials and methods

Patients with a diagnosis of differentiated metastatic thyroid cancer (stage 2 for patients younger than 55 years, or stage 4 for patients aged 55 years or older, according to 8th edition of the AJCC/TNM manual) [17], refractory to radioactive iodine treatment, in therapy with lenvatinib, regularly followed at the Translational Precision Medicine Unit of Policlinico Umberto I in Rome between November 2011 and September 2021, were retrospectively enrolled in the study. Eight patients were excluded from the study because of drug discontinuation due to intolerable toxicities, follow-up evaluation time shorter than one month or drug interruption at the time of follow-up visit.

Clinical and anthropometric characteristics were collected for both the day of therapy initiation (T0) and the follow up (T1) from electronic medical records. Patients did not change the baseline lipid- or glucose-lowering therapy during the follow-up time.

Laboratory determination was carried out on the frozen plasma samples collected at T0 and T1, obtained from patients who were fasting at the time of sample collection, and stored with informed consent. The determination of lipid profile (total cholesterol [TC], triglycerides [TRG] and HDL cholesterol [HDL-C]), glucose, liver enzymes (AST, ALT, GGT), creatinine and total proteins was carried out enzymatically, with the automatic analyzer *ILab 300 plus* (Werfen Instrumentation Laboratory, Lexington, MA). LDL cholesterol (LDL-C) was calculated through the Friedwald’s formula when TRG levels were <400 mg/dl.

Severity of glucose and lipid alterations was classified according to the Common Terminology Criteria for Adverse Events (CTCAE) classification version 5.

The SCORE2 [18] or SCORE2-OP [19] risk-prediction algorithms were used to estimate 10-year risk of cardiovascular disease (at baseline and follow up) in patients younger and older than 70 years, respectively. For each patient the difference between the LDL-C level and the optimal LDL-C target according to the SCORE2/SCORE2-OP was calculated for both T0 and T1. Given the potentially shorter survival of this specific cohort, the 5-year estimation of cardiovascular risk was also performed using the Australian cardiovascular disease risk calculator (Aus CVD Risk Calculator). [20]

Data from CT scans carried out as part of the regular follow up program was used to assess the tumor burden change from T0 to T1 according to the Response Evaluation Criteria in Solid Tumors (RECIST). We evaluated CT scans that were performed in a window of time of maximum one month before or after the blood sampling. Tumor response was then classified as: progressive disease (PD), stable disease (SD) partial response (PR), or complete response (CR).

For each category of tumor response, the difference between total cholesterol (ΔTC), triglycerides (ΔTRG) and LDL-C (ΔLDL-C) from T1 to T0 was calculated.

Statistical analysis of the results was performed with IBM SPSS Statistics version 26 software. Kolmogorov–Smirnov test was used to assess parametric or non-parametric distribution of variables. Paired *t* Student test and Mann–Whitney test were used to evaluate mean difference between paired parametric and non-parametric variables respectively. McNemar test was used to compare the difference between paired categorical variables and Kruskal–Wallis test to study mean differences among more than 2 groups. Statistical significance was defined as a *P* value < 0.05.

This study was approved by the ethics committee of Policlinico Umberto I, Rome (approval number: 4798).

## Results

### Baseline characteristics of the population

Twenty-nine patients were included in the final study cohort. Baseline characteristics of the population are described in Table 1. Patients had a median age of 63 years and the majority (62%) were males. At baseline, mean BMI was 28 Kg/m², sixty-two percent of patients had already a diagnosis of hypertension and all the subjects enrolled had no previous cardiovascular events.

**Table 1:** Baseline characteristics of the study population

Variable	
Age (years)	63 (40–86)
Sex (%M)	62
BMI (Kg/m²)	28 (±5)
Smokers (%)	17
Previous CVD (%)	0
Anti-hypertensive therapy (%)	52
Glucose-lowering therapy (%)	7
Lipid-lowering therapy (%)	7
Cancer histotype	
papillary thyroid cancer (%)	55
follicular thyroid cancer (%)	17
poorly differentiated thyroid cancer (%)	21
oncocytic thyroid cancer (%)	7

Parametric variables are expressed as mean (±standard deviation); Non-parametric variables are expressed as median (minimum-maximum)

*CVD* cardiovascular disease

Median follow up time was 10 months (1–15 months). Twenty-one patients had a follow up at 12 ± 6 months, while eight patients had a follow up at 1–6 months from baseline. For the second group of patients, the median follow up time was 3.5 months (1–5 months).

### Variables of interest

**Fig. 1:**
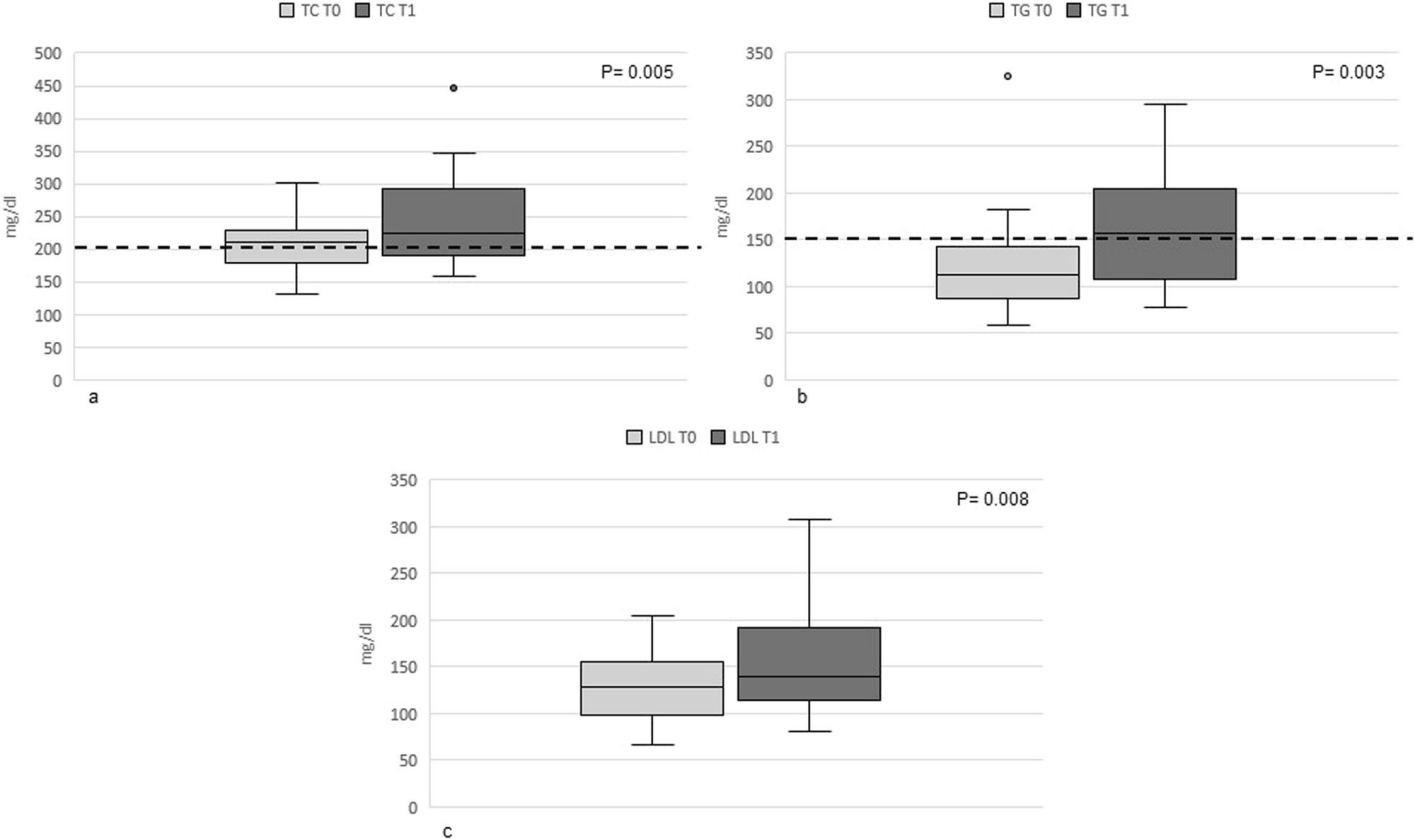
Levels of TC (**a**), TG (**b**) and LDL-C (**c**) before and after starting lenvatinib (dotted line representing the normal cutoff value)

Changes in the variables of interest from T0 to T1 are described in Table 2. A statistically significant decrease in both BMI and Lenvatinib dose prescription from baseline to follow up was observed. No statistically significant difference was observed in in-clinic blood pressure values, probably due to an implementation of anti-hypertensive therapy in the majority of patients during follow-up. The prevalence of lipids and glucose adverse events at T1 is shown in Table 3. A statistically significant increase in TC, TRG and calculated LDL-C was observed from baseline to follow up (Fig. 1). No differences in HDL-C, glucose and TSH levels were observed.

**Table 2:** Changes in the variables of interest

Variable	T0	T1	*P* value
BMI (Kg/m²)	28 (±5)	26 (±4)	**0.003**
Systolic BP (mmHg)	135 (110–185)	140 (100–175)	0.754
Diastolic BP (mmHg)	80 (70–110)	85 (60–100)	0.784
Anti-hypertensive therapy *n* (%)	15 (52)	26 (90)	**0.001**
Lenvatinib prescribed dose (mg/die)	20 (10–24)	14 (4–24)	**0.028**
HDL-C (mg/dl)	54 (±11)	57 (±17)	0.335
Glucose (mg/dl)	111 (73–210)	111 (80–155)	0.307
TSH (UI/L)	0.360 (0.003–8.400)	1.035 (0.030–37.140)	0.058

Parametric variables are expressed as mean (±standard deviation); Non-parametric variables are expressed as median (minimum-maximum). Bold highlights a statistical significant difference.

**Table 3:** Prevalence of lipids and glucose alterations and severity according to CTCAE criteria in patients on treatment with lenvatinib at T1

Event	Grade 1–2	Grade 3–4
Hypercholesterolemia *n* (%)	1 (3)	1 (3)
Hypertriglyceridemia *n* (%)	10 (34)	0 (0)
Hyperglycemia *n* (%)	4 (14)	0 (0)

### Cardiovascular risk

**Fig. 2:**
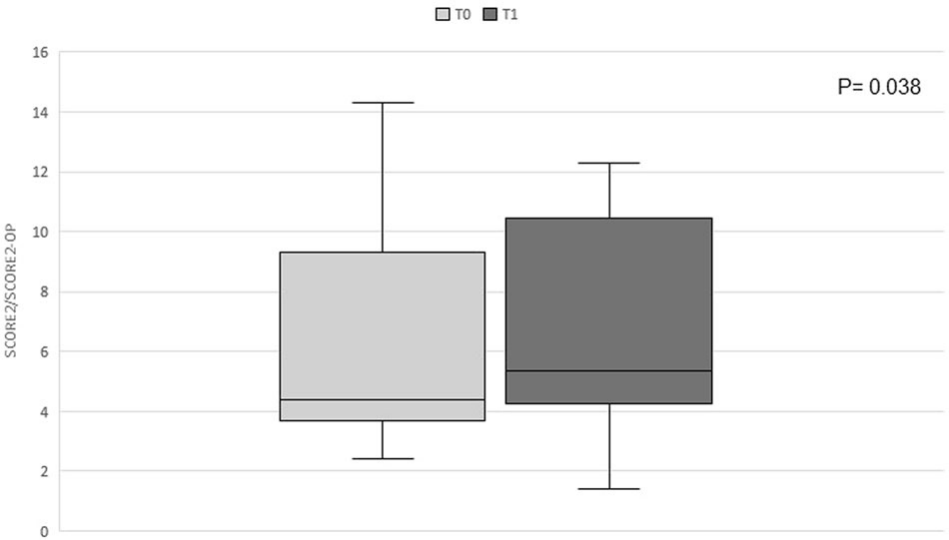
Change in SCORE2/SCORE2-OP before and after starting lenvatinib

A statistically significant increase in the SCORE2/SCORE2-OP from baseline to follow up was observed, as shown in Fig. 2. Since blood pressure values did not vary significantly between T0 and T1, the increase in SCORE2 is likely due to the increase in TC and LDL-C. A statistically significant increase in the 5-year risk of cardiovascular disease according to the Australian cardiovascular disease risk calculator from baseline to follow-up was observed as well (5.5 ± 3 vs 6.5 ± 4, *P* value 0.023).

### Distance to target LDL-C

**Fig. 3:**
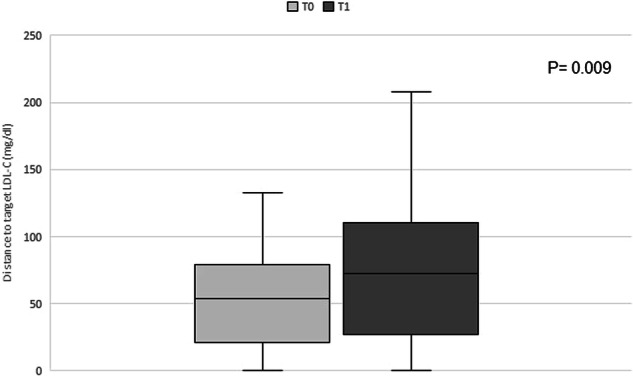
Changes in distance to “optimal” LDL-C target before and after starting lenvatinib

In the majority of patients an increase in distance to optimal LDL-C target was observed as shown in Fig. 3. Mean distance to the desired LDL-C target increased from 53.2 (±37) to 78.5 (±57) from T0 to T1 (*P* value 0.009).

### Metabolic adverse events and tumor response according to the Response Evaluation Criteria in Solid Tumors (RECIST)

**Fig. 4:**
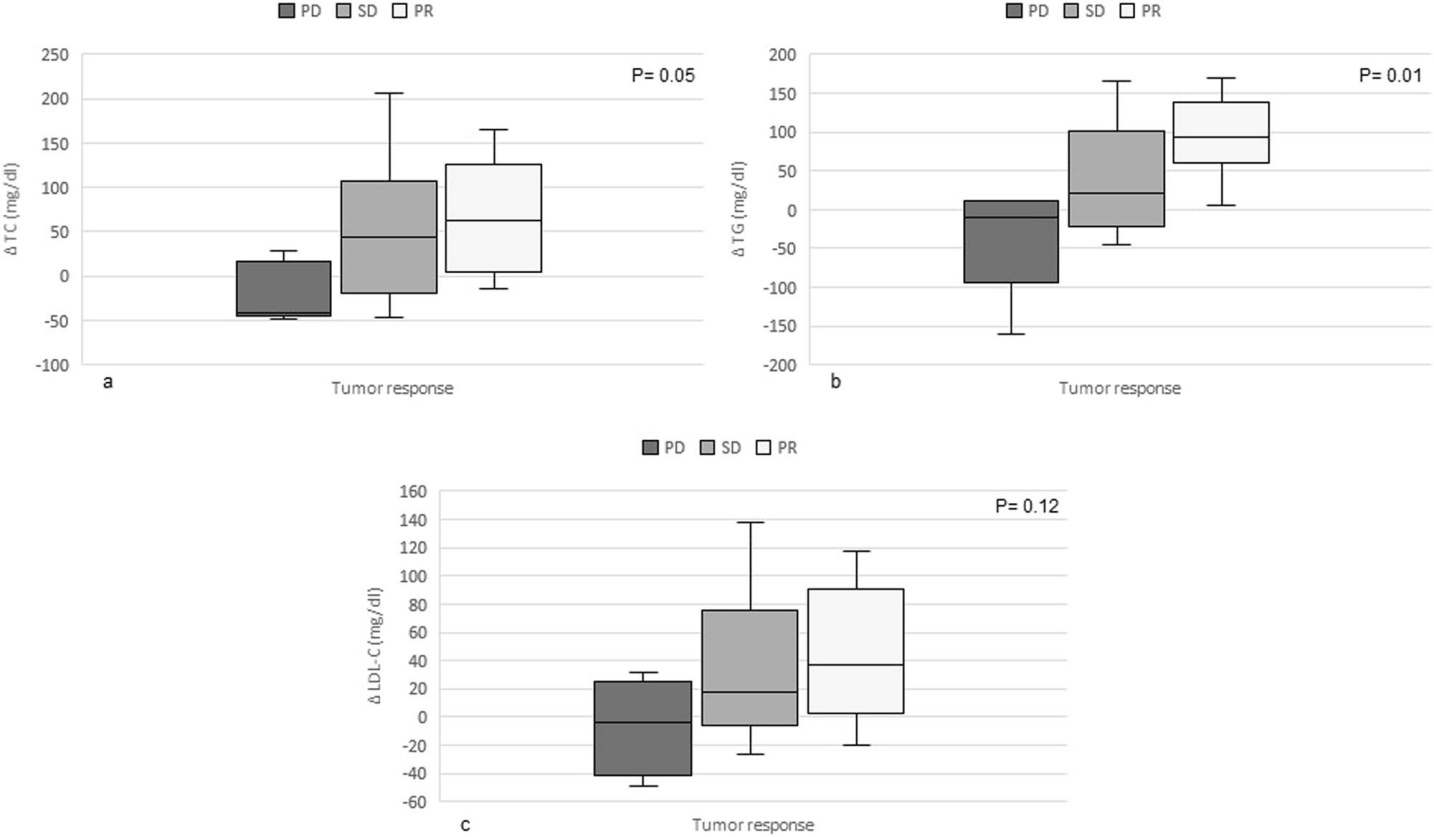
Relationship between tumor response and variation in TC (**a**), TG (**b**) and LDL-C (**c**) levels

The relationship between the change in tumor burden according to the RECIST criteria and the difference in total cholesterol (ΔTC), triglycerides (ΔTRG) and LDL-C (ΔLDL-C) between T1 and T0 is depicted in Fig. 4. As expected, the majority of patients showed a positive response to therapy (either stability or partial response). It has been observed that TC, LDL-C and TRG increase progressively with increasing response to therapy. Median daily lenvatinib dose did not vary significantly among the three groups (PD: 14 mg/dl [10–24]; SD: 12.2 mg/dl [8–24]; PR: 20 mg/dl [4–20]; *P* value 0.79). SCORE2/SCORE2-OP and distance to target LDL-C did not change significantly between T1 and T0 according to tumor response to therapy.

## Discussion

Analysis of data showed no effect on glucose levels. Grade 1–2 hyperglycemia was observed in 4 patients, but no significant difference was observed in median blood glucose levels from T0 to T1.

A worsening of the lipid profile of the subjects treated with lenvatinib was documented. In particular, the most commonly observed adverse event was a moderate increase in triglyceride levels from baseline to follow up, classifiable as grade 1-2 according to the CTCAE classification system version 5 (corresponding to serum triglyceride levels between 150 and 500 mg/dL). Median triglyceride levels increased significantly, despite a significant decrease in BMI values. No grade 3 or superior hypertriglyceridemia (>500 mg/dL) was observed. Hypercholesterolemia was observed in a minority of patients, but it was more pronounced, with one patient developing grade 3 hypercholesterolemia (serum total cholesterol levels between 400 and 500 mg/dL), associated with a decrease in BMI and lenvatinib dose from T0 to T1. Accordingly, an increase in 10-years estimated risk of cardiovascular disease mainly driven by an increase in LDL-C from T0 to T1 was observed as well. Given the debatable clinical significance of a long-term estimation of cardiovascular disease risk in patients with metastatic cancer, we conducted a shorter (5-year) risk assessment using the Australian cardiovascular risk calculator. Our findings demonstrated an increase in the 5-year risk of cardiovascular disease as well.

Pathophysiology underlying lipid alterations associated with MKIs at present remains unclear. Since TSH levels did not vary significantly, hypothyroidism was likely not the cause of the increase in lipid levels observed in the study population; moreover, the only patient in our population with TSH levels equal to 37.140 UI/L at T1 had normal total cholesterol, LDL-C and triglyceride levels. It has been suggested that some MKIs possibly affect lipid metabolism by acting on platelet-derived growth factor, that has a synergistic action with LDL-receptor-related-protein (LRP) [21, 22].

As expected, most patients showed clinical benefit (either stability of the disease or partial response to therapy), with only a minority of subjects experiencing progressive disease. Interestingly, our results suggest a relationship between alterations of the metabolic profile and patients’ response to therapy, within a certain degree of variability. Various studies suggest that on-target adverse events indicate effective inhibition of receptors involved in cancer progression, leading to better outcomes [23]. Zheng et al. found a positive correlation between the number and severity of adverse events and tumor response in patients with metastatic renal cell carcinoma treated with sorafenib [24]. To our knowledge, no study in the scientific literature investigated the association between metabolic adverse events and change in tumor burden in patients treated with MKIs. It is known that the VEGF family has a role in the regulation of intestinal absorption of lipids [25], lipoprotein lipase (LPL) [26], and intracellular lipid metabolism [27]. If these effects will be confirmed in future studies, it could be useful in paving the way for a deeper understanding of still unknown causes of dyslipidemia. Furthermore, these effects may be considered surrogate markers for drug antitumoral activity or bioavailability. In any case, although in these patients the underlying oncological condition is the most frequent cause of death, the fact that metabolic adverse events are more likely in patients with clinical benefit highlights the importance of preventing cardiovascular events.

While these results are preliminary in nature, available data on the prevalence and severity of metabolic alterations in patients treated with MKIs is limited, as pointed out in our recent systematic review [15]. The majority of evidence about metabolic adverse events of MKIs are reported in randomized controlled trials, while little data from observational studies of real-life clinical practice is available. Furthermore, many studies are difficult to interpret, since they evaluate patients treated with combinations of antineoplastic drugs, or report only severe (grade 3-4) metabolic adverse events [15].

This study had some limitations. In the first place, since advanced thyroid cancer is a rare tumor, the number of patients recruited is small and thus probably not fully representative of the entire population. Moreover, the length of follow up is overall short and differs among patients. Even if metabolic adverse events are usually observed after 1 month of treatment [1, 28], having different follow up times is a source of heterogeneity. Additionally, it was not possible to include patients treated with other MKIs due to lack of available data, even though metabolic alterations appear to be reported with other molecules as well [15]. Being this a retrospective study, it was also not possible to include a control group, nor to gain information about lifestyle and diet of patients before and during therapy, which could have had a confounding role in the alteration of the metabolic profile. Furthermore, the study utilized a scoring system (SCORE2 and SCORE2-OP) designed to assess 10-year cardiovascular risk, although survival periods in some cases were shorter. Notably, validated scoring systems for evaluating shorter-term cardiovascular risk specific to the European population were unavailable [29]. These findings underscore the need for developing more appropriate and shorter-term cardiovascular risk assessment tools for oncology patients, which could also address their varying survival rates.

In conclusion, this study found a significant association between the use of lenvatinib and the development of lipid alterations in patients with advanced thyroid cancer. These metabolic alterations appear to be related to both an increase in cardiovascular risk and a better RECIST response to therapy. In our opinion, when dealing with antineoplastic therapies that potentially increase patients’ life expectancy, metabolic adverse events should be considered and periodically assessed, in order to detect all therapy-related cardiovascular toxicities and control their potential morbidity and mortality [16, 30]. Future research should focus on developing more appropriate management strategies, including specific monitoring and management protocols for metabolic side effects, also taking into account possible pharmacological interactions. Additionally, enhancing awareness on this issue could lead to early detection of metabolic alterations and further improve patient outcomes. Moreover, understanding the reason behind the development of worse metabolic AEs in a minority of patients, may be useful for both a more adequate clinical surveillance and to potentially understand still unknown mechanisms involved in their pathogenesis.
